# Anemia in Inflammatory Bowel Disease: An Under-Estimated Problem?

**DOI:** 10.3389/fmed.2014.00058

**Published:** 2015-01-19

**Authors:** Gerhard Rogler, Stephan Vavricka

**Affiliations:** ^1^Division of Gastroenterology and Hepatology, University Hospital Zürich, Zürich, Switzerland; ^2^Division of Gastroenterology, Triemlispital, Zürich, Switzerland

**Keywords:** anemia, inflammatory bowel disease, iron desorption

## Abstract

Anemia is one of the most frequent complications and/or extraintestinal manifestations of inflammatory bowel disease (IBD). Iron deficiency is the most important cause of anemia in Crohn’s disease and ulcerative colitis patients. Iron deficiency even without anemia may impact the quality of life of our IBD patients. In the last 10 years, the understanding of the pathology of iron-deficiency anemia and “anemia of chronic diseases” has increased; new diagnostic tools have been developed and new therapeutic strategies have been discussed. Hepcidin has been identified to be a central regulator of iron absorption from the intestine and of iron plasma levels. Hepcidin is regulated by iron deficiency but also as an acute phase protein by pro-inflammatory mediators such as interleukin-6. Innovative diagnostic tools have not been introduced in clinical routine or are not available for routine diagnostics. As iron substitution therapy is easy these days with a preference for intravenous substitution, the impact of differential diagnosis of anemia in IBD patients is underestimated.

## Anemia is a Frequent Problem in IBD Patients

Anemia represents the most common systemic complication and/or extraintestinal manifestation in inflammatory bowel disease (IBD) (1, 2). A recent systematic review of relevant manuscripts published between 2007 and 2012 reported a prevalence of anemia in patients with Crohn’s disease of 27% (95% confidence interval, 19–35) and of 21% (95% confidence interval, 15–27) in patients with ulcerative colitis (2). More than half of the anemic patients (57%) were found to be iron deficient (2). Others report a prevalence of anemia in IBD patients ranging between 6.2 and 73.3% (3).

In IBD outpatients, the frequency of anemia appears to be lower (16%) as compared to hospitalized patients (68%) (3). Anemia prevalence in hospitalized IBD patients ranges from 44% as reported by Hoffbrand et al. (4) up to 74% as found by Werlin and Grand (5). However, the latter numbers were reported in older studies from the 60s and 70s. The prevalence has decreased as obvious from newer investigations and meta-analyses, but the overall progress we have made in improving treatment of anemia and outcome is surprisingly small.

Some studies have found a correlation between high Crohn’s disease activity index (CDAI) and anemia; however, this is not surprising as the hematocrit value is one parameter for the calculation of the CDAI. Therefore, CDAI and hematocrit/hemoglobin levels are no independent parameters and should not be analyzed as independent variables. On the other hand, it has been repeatedly described that anemia as well as iron deficiency significantly impair the quality of life in patients with chronic intestinal inflammation (6). In the FERGIcor study, a randomized controlled trial on ferric carboxymaltose for iron-deficiency anemia (IDA) in IBD patients disease-related (IBDQ) and mental quality of life (SF-36) improved by iron supplementation during the 12-week-study period (7). Similar findings on the quality of life are reported from other iron supplementation studies (8, 9). On the other hand, being anemic or iron deficient was not associated with increased fatigue in a Scandinavian cohort (10).

A successful therapy of anemia may even improve the quality of life better than the therapy of disease activity (anti-inflammatory therapy) (6). Further, anemia was even found to be among the most frequent comorbid conditions associated with death in IBD patients (11). However, in those patients, it may simply reflect the severity of the underlying IBD.

In the last decades, the incidence of anemia in IBD patients has been reported to decrease (3). This observation is probably related to improvements in treatment of these diseases or in iron supplementation. On the other hand, our recent study indicated that treating gastroenterologists may still accept the concept of “asymptomatic anemia,” claiming that patients would adapt to low hemoglobin levels if anemia developed slowly as this is typically the case in IBD (12).

**Figure 1:**
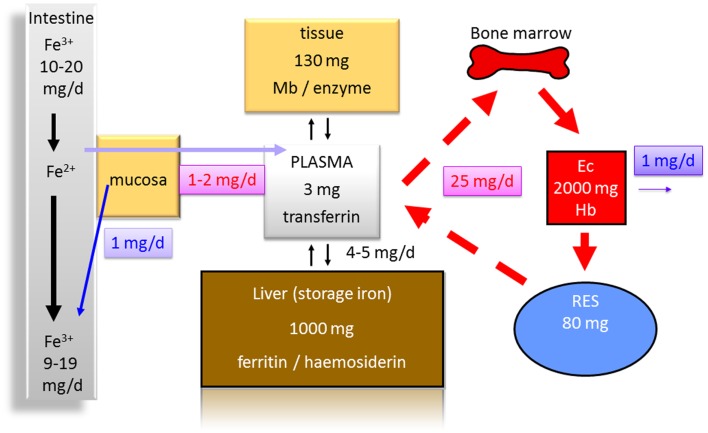
**Distribution of iron in the human body (70 kg)**. Less than 10% of the daily iron uptake of around 20 mg is absorbed by the intestinal mucosa. The transferrin bound iron pool in the plasma consists of about only 3 mg in total. Around 1000 mg are bound to ferritin and hemosiderin as iron storage in the liver. One hundred to hundred and thirty milligrams are found in other tissues [bound to myoglobin (Mb) or to enzymes]. Erythrocytes (Ec) contain about 2000 mg iron in total bound to hemoglobin (Hb). Eighty milligrams are found in the reticulo-endothelial system (RES) in which senescent erythrocytes are degraded. From the RES, iron is released into the serum contributing to the pool bound to transferrin. Around 1 mg of iron is physiologically lost each day contributing to a balance of iron metabolism and making a higher uptake in the intestine normally unnecessary.

Several reasons for anemia in IBD patients exist. Iron deficiency (Figure 1), vitamin B12 deficiency, and anemia of chronic diseases are certainly the most important. Iron deficiency is found frequently in IBD patients and ranges from 36 to 90% with a mean prevalence of 45% (3).

When we looked into patient data of a cross-sectional cohort in Switzerland (the Swiss IBD Cohort Study), we found anemia in 12.9% of IBD patients in private practice, and 28.8% of IBD patients in tertiary centers (12). Whereas this difference was statistically significant, the frequency of iron deficiency was not different between patients treated in university hospitals from patients in private practice (12). As expected in this cohort-based study, a higher CDAI was correlated with lower hemoglobin levels (12).

## Iron Deficiency in IBD Patients

Iron deficiency still has a high prevalence in patients with IBD, which is not always paid sufficient attention to (13). In a population-based cohort of 279 patients (183 Crohn’s disease, 90 ulcerative colitis, and 6 indeterminate colitis) in Germany, Ott and coworkers found an IDA in 26 of 68 patients with anemia (38.2%), but only 9 patients received subsequent iron therapy (13). The numbers did not change a lot after 1 year as still 27 patients were identified to have an IDA, however, 71.4% of them were treated (13). 54.3% did not receive iron substitution despite IDA (13). This indicates that there is still a high prevalence of iron deficiency in the IBD population, which does not change a lot after the diagnosis is established and that frequently iron deficiency is treated insufficiently.

Two major reasons for iron deficiency associated with IBD have to be considered. One is an impaired uptake (Figure 1), e.g., due to functionally disturbed absorption caused by inflammation of the ileum. An impaired absorption also may occur in patients with severe ulcerative colitis. If patients have more than 20 bowel movements per day and report that food appears in the rectum 30 min after uptake, it is easy to imagine that a sufficient absorption time in the small bowel is not present (14, 15). This is also indicated by the fact that patients with severe ulcerative colitis may have reduced serum protein and albumin levels indicating a malabsorption. An additional mechanism that antagonizes iron absorption in the gut is discussed below. The second major reason for an iron deficiency in IBD certainly is the continuous blood loss in active colitis or ileitis associated with a depletion of iron and iron stores (Figure 1).

As iron is an essential component of heme and hemoglobin, it is obvious that malabsorption of iron or continuous loss of iron may restrict iron delivery to erythrocyte precursors (Figure 1) and subsequently limits erythropoiesis (16, 17).

Iron deficiency may already impact the quality of life without anemia (16, 18–20). This may be partially caused by non-hematological effects of iron-deficiency (21, 22); however, the impact, for example, on myoglobin function is somewhat controversial.

In the past, the diagnosis of IDA was based on traditional biochemical markers such as serum iron, transferrin saturation (TfS), and ferritin (23–25). Better parameters to identify iron deficiency such as soluble transferrin receptor (sTfR) are not applied widely (26). If the “traditional” markers appeared to be in a normal range and there still was an anemic blood count in IBD patients, this was attributed to “anemia of chronic disease” (24, 27–29). Consecutively, this was a diagnosis of exclusion and no positive criteria were used.

In the last 10 years, the understanding of the pathophysiology of anemia has made huge progress and this should change our diagnostic procedures in IBD patients and finally modify treatment strategies. A very important regulator of iron homeostasis, centrally involved in iron absorption is hepcidin (30). It not only determines the rates of absorption but also influences plasma iron levels and iron distribution (30). Hepcidin is produced and secreted mainly by hepatocytes, and to a lesser extent by macrophages and adipocytes (30). To fully understand the reasons for iron deficiency in IBD patients, the function of hepcidin has to be addressed.

## Role of Hepcidin for Iron Metabolism in IBD Patients

**Figure 2:**
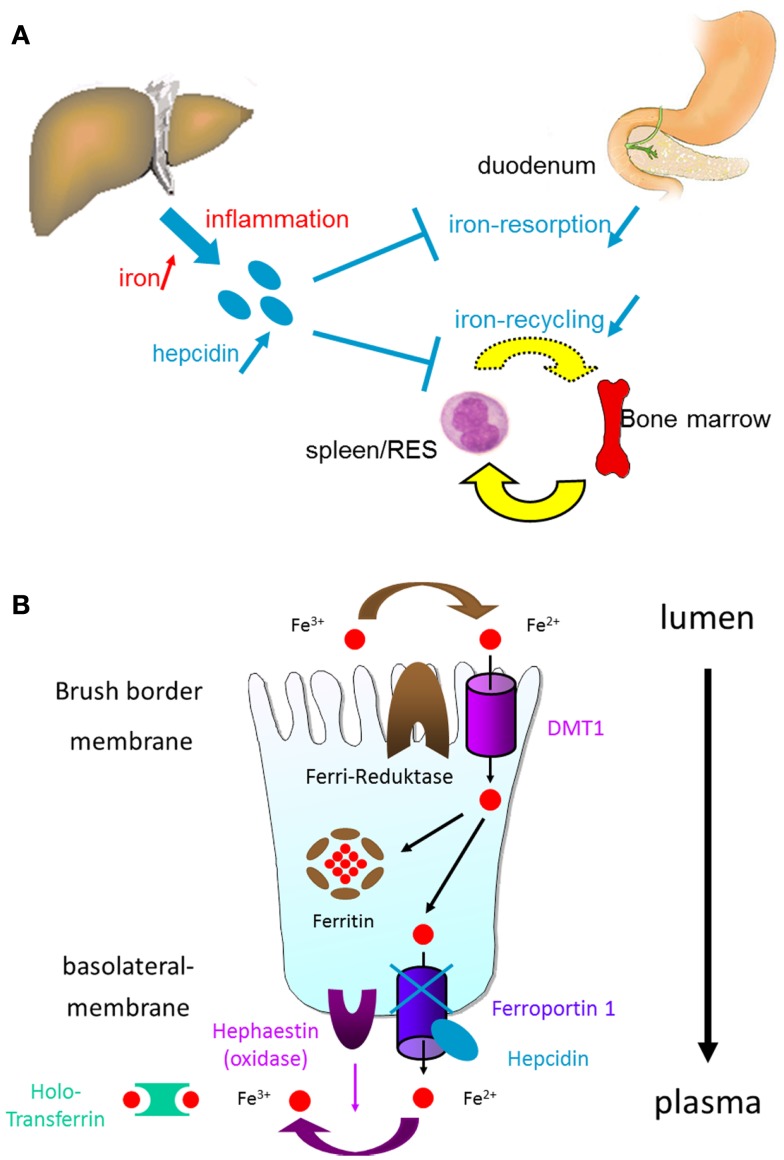
**(A)** Regulation of iron absorption by hepcidin. Hepcidin synthesis is increased upon iron or inflammation in the liver. Inflammation in the gut will lead to increased IL-6 serum levels followed by higher amounts of hepcidin in the circulation. Hepcidin then decreases iron absorption in the gut and iron recycling from the RES leading to reduced iron availability for hem formation in the bone marrow. **(B)** On a molecular level in the enterocytes – the absorptive cells of the intestinal brush border membrane – hepcidin prevents iron release from enterocyte cytoplasmic stores by binding to the iron exporter protein ferroportin. This is followed by ferroportin internalization and degradation. Thus, iron is trapped intracellulary without being able to reach the blood stream (DMT1, divalent metal transporter 1 is a protein by the SLC11A2 gene (solute carrier family 11, member 2). DMT1 is a metal ion transporter protein that binds a variety of divalent metals including copper (Cu^2+^) and zinc (Zn^2+^) but is best known for transporting ferrous iron (Fe^2+^).

The protein hepcidin was first described in 2000 as a member of the anti-microbial protein family of defensins (31, 32). Besides the site of synthesis (“hep”) and the anti-microbial properties (“cide”) that occur in the name hepcidin has important further functions (30, 33, 34). Under physiologic conditions, hepcidin inhibits iron flow into plasma from macrophages that have taken up and degraded senescent erythrocytes as well as their hemoglobin, and also from small intestinal epithelial cells involved in the absorption of dietary iron (35–38) (Figure 2A). This iron release from cytoplasmic stores is prevented by binding of hepcidin to the iron exporter protein ferroportin (39–41) (Figure 2B). Ferroportin is internalized and degraded upon hepcidin activity (Figure 2B). This mechanism of ferroportin degradation requires ubiquitination that is induced or triggered by hepcidin (39, 40). As ferroportin is the most important protein to liberate iron from cytoplasmic stores, a degradation of ferroportin must reduce the iron available for erythropoiesis (34, 42–44) (Figure 2B). In small intestinal epithelial cells, this would mean that iron is “locked” in the cytoplasmic stores without a chance to enter the plasma and subsequently circulation where it finally may arrive in the bone marrow for erythropoiesis (Figure 2B). The retention of iron in the small intestinal epithelial cells also means a loss of this iron pool as the enterocytes will be exfoliated during their normal life cycle within 5 days finally leading to loss of this “trapped” iron for the body.

Hepcidin synthesis is regulated by iron and erythropoietic activity (45, 46) (Figure 2A). Hepcidin expression is down-regulated in anemia with concomitant iron deficiency (46). Thus, ferroportin is not internalized from the cell membrane and iron can be transported from stores to the plasma to provide heme synthesis and erythropoiesis with more iron (Figure 2B). Iron deficiency is associated with reduced bone morphogenetic protein-6 expression and impaired SMAD1/5/8 phosphorylation, which are key events for hepcidin transcription and expression (45, 46). On the other hand during anemia of chronic diseases (without significant iron deficiency), hepcidin expression is even increased, which may explain why iron is “trapped” intracellularly under these conditions (46) (Figure 2A). As obvious, sensing of iron levels and an early detection of an iron deficiency is of crucial importance.

Plasma iron levels are sensed and “measured” by two transferrin receptors (TfR1 and TfR2) (47–49). Both receptors transmit the information on plasma transferrin levels to intracellular signaling pathways that among other cellular reactions also regulate hepcidin synthesis (50, 51). High plasma iron levels and high iron stores or even “iron overload” will stimulate hepcidin synthesis, which then inhibits iron absorption from the intestinal lumen. In contrast, iron deficiency and increased erythropoietic activity suppress hepcidin transcription and translation, decreasing its synthesis. Subsequently, in anemic IBD patients in remission with iron-deficiency, low hepcidin levels have been reported (30, 52–57). The low hepcidin normally would allow increased absorption of dietary iron and release of iron from stores, which may not always be possible in IBD patients. Why do patients with IBD sometimes show increased hepcidin levels despite an iron deficiency?

In IBD (similar to other chronic inflammatory disease or infections), it can be assumed that inflammation increases hepcidin expression and synthesis as hepcidin also is an “acute phase protein” (38, 45). This induction of hepcidin protein synthesis is at least partially mediated by interleukin-6 via its receptor and subsequent intracellular signal transduction via signal transducer and activator of transcription-3 (STAT3) (45, 52, 53, 58–60). Other cytokine-mediated pathways also may play a role. Increased hepcidin levels in patients with IBD and other inflammatory diseases cause the retention of iron in macrophages and enterocytes, leading to hypoferremia and iron-restricted erythropoiesis (53). In a recent study by Basseri and coworkers, a positive correlation between serum hepcidin and IL-6 levels in IBD patients was confirmed (53). The authors further detected a strong negative correlation between serum hepcidin concentrations and hemoglobin levels (*p* = 0.029) (53). The authors conclude that anemia of chronic diseases is associated with high serum IL-6 and hepcidin levels, which supports the hypothesis that IL-6-driven hepcidin production mediates anemia in patients with IBD (53). With this in mind, the new therapeutic anti-IL-6 strategies tested in clinical trials in patients with Crohn’s disease may be of special interest also for anemia of chronic diseases in IBD.

In contrast, when serum hepcidin levels were investigated from 247 IBD patients (130 CD and 117 UC) from the Swiss Inflammatory Bowel Disease Cohort Study (SIBDCS), we found that independent of inflammatory activity, all patients with decreased ferritin had significantly lower hepcidin levels (55). Further, a significant correlation between serum ferritin levels and serum hepcidin was found (55). In this cohort by applying a backward multi-linear stepwise regression analysis only ferritin, but none of the inflammatory markers correlated significantly with hepcidin concentration suggesting that iron deficiency was the key trigger for hepcidin regulation (55). Thus, depending on the IBD population, an acute phase reaction may play a role or not for hepcidin regulation, iron deficiency, and subsequent anemia.

## Should We Change Our Diagnostic Routine with Respect to Iron Deficiency and Anemia in IBD Patients?

As mentioned, the laboratory evaluation of anemia and iron deficiency in IBD patients has traditionally been based on low serum iron, low TfS, and low ferritin. However, those commonly used laboratory tests as well as total iron-binding capacity and mean corpuscular volume provide only limited diagnostic value in patients with chronic active IBD or in patients presenting with acute flares of the IBD in whom we do not have recent determinations of the discussed parameters. The acute phase reaction will definitely change the ferritin values determined. Inflammation can mimic some aspects of iron deficiency by impairing the utilization of existing iron stores for red cell production as explained in detail above. The pathophysiological mechanism responsible for the reduced availability of iron from its cytoplasmic stores may be caused by cytokine stimulated hepcidin expression and subsequent ferroportin degradation.

Serum ferritin has the potential to differentiate true iron deficiency from inflammatory iron sequestration. However, ferritin determination has its limitations as it is an acute phase protein and may show false high levels in patients with low iron stores due to an acute flare of the underlying IBD (61). Both inflammation and intracellular iron accumulation can stimulate the production of ferritin. Therefore, interpretation of serum ferritin in patients with inflammation is tricky (55, 62, 63).

The differentiation among absolute iron deficiency, functional iron deficiency, and iron-sequestration syndromes (leading to “anemia of chronic diseases”) is important for patient management (61).

Increased serum levels of sTfR protein have been reported to be a good indicator of iron deficiency (24, 26, 48, 61, 63–65). sTfR is released in proportion to the expansion of erythropoiesis in the bone marrow and is not regulated in its expression or secretion by inflammation (24, 26, 63, 66). However, this assay has not made it into clinical routine as it was found that it only can provide a specificity of 84% and a positive predictive value of only 58% in a population with diagnostically difficult situations (67). Several indices have been calculated and evaluated for their diagnostic accuracy, such as the combination of ferritin and sTfR (sTfR/log ferritin) (68–70). However, still diagnostic sensitivity and specificity are not completely satisfactory when inflammatory conditions are present as in IBD patients.

Recent flow cytometry-based assays seem to improve the diagnostic repertoire. The flow cytometric analysis of reticulocytes allows determination of reticulocyte hemoglobin (CHr) content or percentage of hypochromic reticulocytes (71). This may be more sensitive than the determination of erythrocyte hemoglobin content or percentage of hypochromic erythrocytes (hypoE) (72). The CHr is sufficiently sensitive to pick up iron-deficient erythropoiesis even in iron-replete volunteers receiving erythropoietin (73). It has been suggested that those reticulocyte indices may allow for real-time evaluation of iron-deficient erythropoiesis and for rapid monitoring of the response to iron substitution therapy (73). An algorithm to screen for iron deficiency including CHr may even increase the accuracy of diagnosis (74).

For a sophisticated differential diagnosis, it may be helpful to quantify hepcidin. Sensitive and accurate immunoassays for human hepcidin have been developed; however, such an assay is not available at many hospitals and even IBD focused care centers even though normal values have been determined.

So what can be recommended as a diagnostic algorithm for iron deficiency and anemia in IBD patients? A basic laboratory screening test should include Hb, full blood counts including reticulocytes, and the assessment of body iron stores including serum ferritin and TfS (17). To evaluate the potential influence of inflammation on serum ferritin C-reactive protein (CRP) as well as calprotectin should be quantified. If anemia is detected further, potential causes have to be evaluated including vitamin B12 and folic acid levels. Haptoglobin, lactate dehydrogenase (LDH), and serum creatinine are useful to exclude potential hemolysis or renal disease, which may exist as extraintestinal manifestation of IBD or as a side effect of medical therapy.

It has been recommended that also patients in clinical remission should be screened every 12 months, whereas patients with active IBD should be screened for anemia every 3 months (17). It has been emphasized that normal hemoglobin levels or normal hematocrit do not exclude iron deficiency.

Reinisch and Munoz pointed to the fact that a normal hemoglobin level with a mean corpuscular hemoglobin (MCH) in the lower limit of normality (normal range: 28–35 pg) or an increased red cell distribution width (RDW, normal range: 11–15) point to mild iron deficiency without anemia (17, 75). RDW seems to be an independent marker of disease activity in IBD patients even without anemia (76).

Iron deficiency in the absence of inflammation (normal CRP and calprotectin) is characterized by low ferritin level <30 ng/mL (1 ng/mL of serum ferritin corresponds to approximately of stored iron). As ferritin is an acute phase protein, normal ferritin levels do not exclude significant iron deficiency in patients with increased CRP or significantly increased calprotectin. In these cases, TfS is preferred and levels below 20% indicate iron deficiency (17).

Reticulocyte Hb content (CHr) may be able to discriminate IDA from anemia of chronic disease (ACD. CHr is reported to have a good sensitivity and specificity for diagnosing iron deficiency and is less affected by inflammation (17, 75).

A low mean corpuscular Hb (MCH <27 pg), or a low reticulocyte Hb content (CHr <28 pg), rather than MCV <80 fL are important markers for IDA. ACD should be suspected when there is evidence of inflammation (as outlined above), anemia, a low TfS (TfS <20%), and normal or increased serum ferritin (>100 ng/ml).

Many patients may present with a combination of IDA and ACD. In those patients, additional laboratory assays can be helpful: hepcidin levels correlate with ferritin and CRP and may replace the ferritin index for confirmation of combined IDA and ACD (hepcidin >4 nmol/L with CHr <28 pg (17, 77).

## Therapy of Iron Deficiency and Anemia in IBD Patients

Oral iron therapy is not well tolerated in many IBD patients. In addition, intravenous iron preparations have proven higher efficacy as compared to oral iron substitution therapy (78–82). The effects of oral iron are difficult to investigate in IBD patients. Therefore, it may be of some interest to look into animal models. Haller and coworkers provided evidence that iron may act as oxidative mediator affecting inflammation at the intestinal surface and inducing cell stress mechanisms (83). Histological analysis of an iron-adequate fed mouse strain (approximately 0.54 mg of iron/day) indeed induced ileal inflammation (83). The impact of oral iron on oxidative stress and intestinal inflammation in the animal model was at least in part mediated by the intestinal microbiota (84). An iron sulfate free diet in combination with systemic iron repletion prevented the development of chronic ileitis in a murine model of Crohn’s disease (84).

Thus, an intravenous therapy for iron substitution is generally preferred in IBD patients. This has been recommended in international expert guidelines: Gasche and colleagues suggest that “the preferred route of iron supplementation in IBD is intravenous, even though many patients will respond to oral iron. Intravenous iron is more effective, better tolerated, and improves the quality of life to a greater extent than oral iron supplements (Grade A).” (85).

Whereas in mild iron deficiency and mild anemia, oral or intravenous iron substitution may be considered (with a clearly faster and higher efficacy of the i.v. route) absolute indications for i.v. iron substitution in IBD patients are severe anemia (hemoglobin <10 g/dL) and disease activation upon, as well as intolerance, or inappropriate response to oral iron (85). Furthermore, severe IBD activity, concomitant therapy with an erythropoietic agent (in severe anemia), or patient preference should be considered (85). In general, dosing and administration intervals depend on the compound.

There are sophisticated formulas to calculate the recommended dosage or iron substitution with i.v. preparation. However, in clinical practice, anemic IBD patients will rarely have a deficit <1000 mg. To avoid iron overload, quantification of TfS may be used. An increase of TfS above 50% may be a useful cut-off to stop therapy and avoid iron overload, especially, as the risk of iron overload can be considered to be very low in patients with active IBD (85).

In severe cases of anemia, erythropoietin or even blood transfusions may become necessary (85). However, these cases are rare and may profit from the involvement of a specialist.

Hepcidin induced by pro-inflammatory mediators such as IL-6 in IBD patients may contribute to the lower efficacy of oral iron substitution therapy in IBD patients (86, 87) leading to ACD (see above).

## How Will the New Insights in Iron Metabolism Impact Future Therapy?

New therapies are under development especially for the treatment of ACD. Some patients with ACD do not respond to anti-inflammatory therapy and even erythropoietin therapy as suggested in the respective guidelines. As in ACD, there is a functional iron deficiency, i.v. iron supplementation in IBD patients may be useful (34). However, iron overload should be avoided.

A new development for the treatment of resistant ACD is monoclonal anti-hepcidin antibodies (34). A humanized anti-hepcidin monoclonal antibody (mAb2.7) was shown to reverse iron restriction and prevent anemia progression in animal models of anemia (34). However, these antibodies may only work in combination with erythropoietin.

As hepcidin is regulated by IL-6 available antibodies that already have been approved for rheumatoid arthritis are presently tested for their effect of hepcidin and ACD.

Another recent development is short interference RNA and anti-sense oligonucleotides against hepcidin (34). The development aims at directly downregulating expression of hepcidin on the mRNA level. Amgen develops a short hairpin RNA (shRNA) strategy against hepcidin, which already was tested in animal models (34). However, only very few anti-sense strategies have found their way into clinical application.

Other agents under development are developed to bind or block hepcidin to inactivate its function.

## Summary

New insights in the pathophysiology of iron deficiency and anemia have been obtained in recent years. Iron deficiency and anemia are important factors for the overall well-being of IBD patients and frequently do not gain the attention they deserve. The diagnostic algorithms have been changed by these new insights as outlined in this overview. Here, parameters are mentioned that are available to most clinicians. Parameters that are very dependent on local conditions and with limited specificity and sensitivity have not been mentioned. The new insights into the pathophysiology of IDA and ACD also slowly change our therapeutic approaches. An integrated care for IBD patients needs to include an up-to-date management of iron deficiency and anemia. Future therapies focused on hepcidin will most likely further improve treatment options and outcome.

## Conflict of Interest Statement

Gerhard Rogler and Stephan Vavricka have received speaker’s honoraria from Vifor.

## Abbreviations

CD, Crohn’s disease; IBD, inflammatory bowel disease; IL, interleukin; NF-kB, nuclearfactor kB; UC, ulcerativecolitis.
